# TLR-4/Notch1/NF-κB pathway modulation by dapagliflozin: a novel mechanism for neuroprotection in hepatic encephalopathy

**DOI:** 10.1007/s11011-025-01681-z

**Published:** 2025-09-08

**Authors:** Hossam H. Abouzaid, Muhammed F. El-Yamany, Yasser O. Mosaad, Mohamed M. Sayed-Ahmed, Riham M. Karkeet, Ayman E. El-Sahar

**Affiliations:** 1 Department of Pharmacology and Toxicology, Faculty of Pharmacy, Cairo University, Cairo, Egypt; 2 Department of Pharmacy Practice and Clinical Pharmacy, Faculty of Pharmacy, Future University, Cairo, Egypt; 3 Department of Cancer Biology, Pharmacology and Experimental Oncology Unit, National Cancer Institute, Cairo University, Cairo, Egypt

**Keywords:** Dapagliflozin, Hepatic encephalopathy, Notch1, TLR-4, Thioacetamide

## Abstract

Acute or chronic liver damage can result in Hepatic Encephalopathy (HE), a potentially fatal neuropsychiatric condition that leads to cerebral and neurological alterations. Dapagliflozin (DAPA), an orally active Sodium/Glucose cotransporter 2 inhibitor with long duration of action. The study aim was to evaluate the potential protective impact of DAPA against HE caused by Thioacetamide (TAA) in rats. HE was achieved *via* a single intraperitoneal TAA dosage of (300 mg/kg). DAPA was administered orally as (1 mg/kg) for 28 days. A total of forty rats were distributed randomly into 4 equal groups: Control (CTRL), Dapagliflozin (DAPA + CTRL), Thioacetamide (TAA), and Dapagliflozin plus Thioacetamide (DAPA + TAA). TAA induced cognitive impairment was alleviated by DAPA, as evidenced by reduction by 63% in escape latency of Morris water maze (MWM) test, elevation in fall off period of Rotarod test, and reduced serum ammonia, Liver enzymes, and restore normal serum albumin levels. DAPA improved the antioxidant capacity and activity of Glutathione by 87.53%; reduced apoptosis, liver necrosis, and astrocyte inflammation. Moreover, DAPA administration reduced gene expression of both Notch1 by 50% and TLR-4 by 55.65% suppressing release of inflammatory cytokines. In conclusion, DAPA possesses a neuroprotective effect, as confirmed by the enhancement of motor incoordination, cognitive deficits, and histopathological alterations. This neuroprotective impact can be justified by lowering hyperammonemia, improving liver functions, in addition to its antioxidant effect, and suppression of TLR-4/Notch1/NF-κB inflammatory pathway.

**Graphical abstract:**

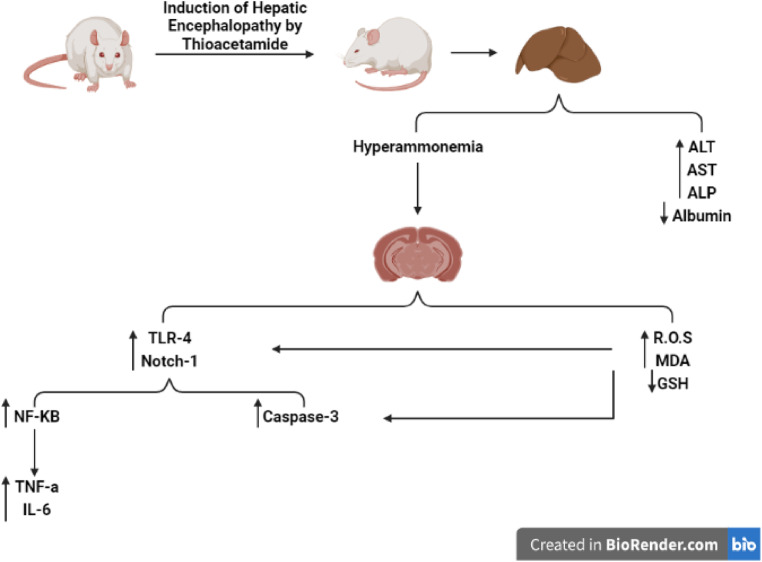

**Supplementary Information:**

The online version contains supplementary material available at 10.1007/s11011-025-01681-z.

## Introduction

Acute or chronic liver failure can result in Hepatic Encephalopathy (HE), a potentially fatal neuropsychiatric condition with mortality rates spinning between fifty to ninety% (Allampati and Mullen 2019). Clinical manifestations of HE include personality changes, inability to pay attention, difficult sleeping, and neuropsychiatric deviations (Gow 2017). Different key-players contributing to the pathogenesis of HE include neurotoxins, inflammation, lessened penetrability of the blood brain barrier, oxidative stress (OS), and bile acid accumulation (Rama Rao and Norenberg 2012; Bosoi and Rose 2013).

Ammonia is a well-recognized influencer of HE as it drives astrocyte inflammation. To maintain homeostasis, nitrogenous substances are metabolized into ammonia *via* the gut microbiome, followed by liver detoxification and renal excretion (Parekh and Balart 2015). Thus, hepatic affection results in hyperammonemia; which bypass the blood brain barrier. Followed by ammonia conversion to glutamine; consequently causing astrocyte inflammation and brain edema (Parekh and Balart 2015).

Toll-like receptors (TLRs) are lipopolysaccharide receptors that perceive both endogenous damage associated molecular pattern molecules (DAMPs) and pathogen associated molecular patterns (PAMPs) triggerring inherent as well as adaptive immunity (Means et al. 2000; Kaisho and Akira 2006). Toll-4 (TLR-4) masters the inflammatory response. It induces NF-κB and its downstream cytokines; causing cellular apoptosis (Makled et al. 2016; Yang et al. 2016). TLR-4/NF-κB pathway is well documented in HE progression (Jayakumar et al. 2014).

Notch (Notch-1) is a membrane receptor that influences cellular differentiation and proliferation, as well as developmental fate switching in different organs. (Arumugam et al. 2018). In the developing brain, Notch signaling act as a neurogenesis inhibitor by hindering the differentiation of the neuroprogenitor cells, while in developed brain it impacts synaptic plasticity as well as learning and memory (Lathia et al. 2008). A clear positive connection is well established between the Notch-signaling pathway and NF-κB pathway, as both pathways manipulate inflammation (Zeng et al. 2017).

Thioacetamide (TAA) a pronounced provoker of hepatic and brain conditions, comparable to human hepatic conditions with neurological symptoms (Lima et al. 2019). The cytochrome P450 breaks down TAA, leading to liver necrosis, OS, and hyperammonemia (Swapna et al. 2006).

Sodium glucose cotransporters (SGLTs) exists in various spots of the central nervous system (CNS) (Yu et al. 2010, 2013; Nevola et al. 2023). SGLT-1 is located in the hippocampal CA-1, CA-3 and dentate gyrus. Additionally, SGLT2 was specifically spotted in the hippocampal tissue, cerebellum, and blood brain barrier endothelial cells (Poppe et al. 1997; Enerson and Drewes 2006; Shah et al. 2012; Jurcovicova 2014).

Sodium/Glucose cotransporter-2 inhibitors (SGLT-2) are oral antihyperglycemics approved for the management of type 2 diabetes mellitus (DM) (Nespoux and Vallon 2020). SGLT2 is most abundantly expressed in the first and second segments of the kidney’s proximal convoluted tubule, where it promotes the re-absorption of glucose from urine by utilizing the sodium concentration gradient (Heise et al. 2013). The primary mode of action of SGLT2 inhibitors involves reducing renal re-absorption of glucose. Thus, these compounds exhibit diuretic, glycosuric, and natriuretic properties, which increase the urine volume and its sodium content (Heise et al. 2013).

Dapagliflozin is usually a tolerated medication. It showed a favourable impact on heart failure patients. Moreover, it decreased the progression of renal disease in patients with atherosclerotic disease (Dhillon 2019). DAPA reported side effects are increased risk of genital as well as urinary tract infections, hypotension, dehydration and diabetic ketoacidosis (Cefalu et al. 2015; Pelletier et al. 2021).

Recently, different research groups documented the antioxidant effects of DAPA (Shihab et al. 2024; Topsakal et al. 2024). DAPA anti-oxidant impact can be attributed to the reduction of reactive oxygen species through activation of sirtuin-1 and the alteration of calcium dynamics (Zaibi et al. 2021; Zhou et al. 2023). Additionally, DAPA increases the activity of superoxide dismutase and glutathione peroxidase activity (Alsereidi et al. 2024).

Along with their anti-hyperglycemic effects, SGLT2 inhibitors have been evidenced to improve hepatic function in diabetic rats (Hazem et al. 2022). Dapagliflozin was proved to enhance Morris water maze performance, maintain the plasticity of synapses within the hippocampus, improve antioxidant capacity, and reduce apoptosis (Sa-Nguanmoo et al. 2017).

Based on the herein mentioned facts, the current study hypotheised and aimed to prove that DAPA could prevent or attenuate HE caused by TAA. Moreover, to investigate the possible molecular pathways behind DAPA neuroprotective effect.

## Results

The dapagliflozin (CTRL + DAPA) group demonstrated no significant difference from the normal group (CTRL) in all assessed parameters.

### Behavioral parameters

**Fig. 1:**
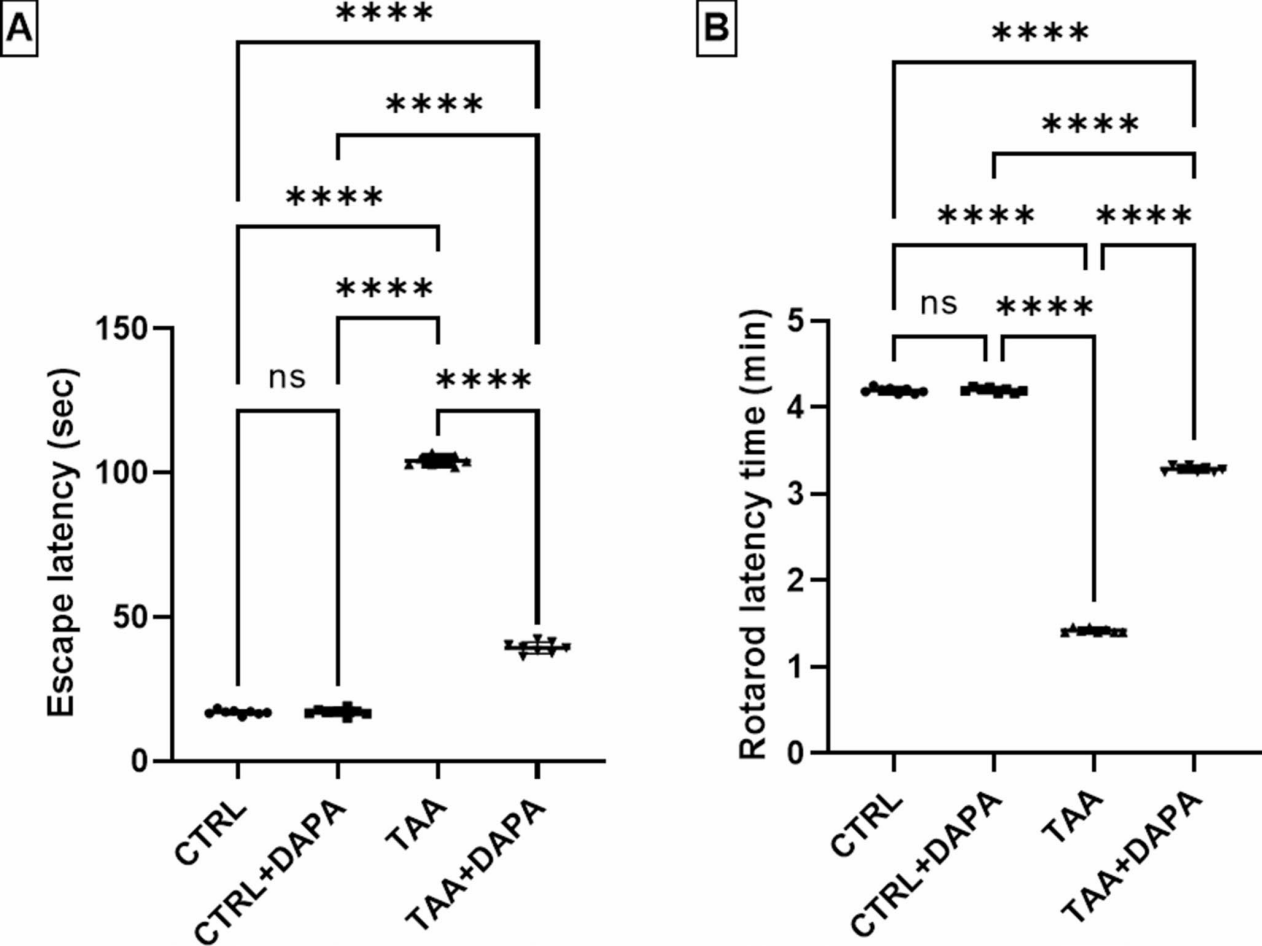
Effect of dapagliflozin (1 mg/kg, p.o.) on (**A**) Morris water maze test, (**B**) rotarod test in TAA-induced (300 mg/kg, I.P.) hepatic encephalopathy in rats. Values are expressed as mean ± SD (*n* = 8). Statistical analysis was performed using one-way ANOVA followed by Tukey’s post-hoc test, with a significant value set at ns: non-significant, * *P* < 0.05, ** *P* < 0.01, ***P < 0.001, and **** *P* < 0.0001

Figure 1 illustrates Morris water maze (MWM) and Rotarod tests used for evaluation of DAPA impact on cognitive defects mediated by TAA. During the MWM, TAA resulted in a significant elevation in escape latency by approximately six folds compared to the control group. Oral administration of DAPA (1 mg/kg) for 28 consecutive days significantly decreased escape latency by 62.97% versus TAA group **(**Fig. 1-A**)**.

Considering Rotarod test, TAA injection significantly decreased the final fall-off time by about three folds compared to the control group. However, oral DAPA dosing normalized the final fall-off time and elevated it to 131.17% versus TAA group **(**Fig. 1-B**)**.

### Serum biochemical analysis

**Fig. 2:**
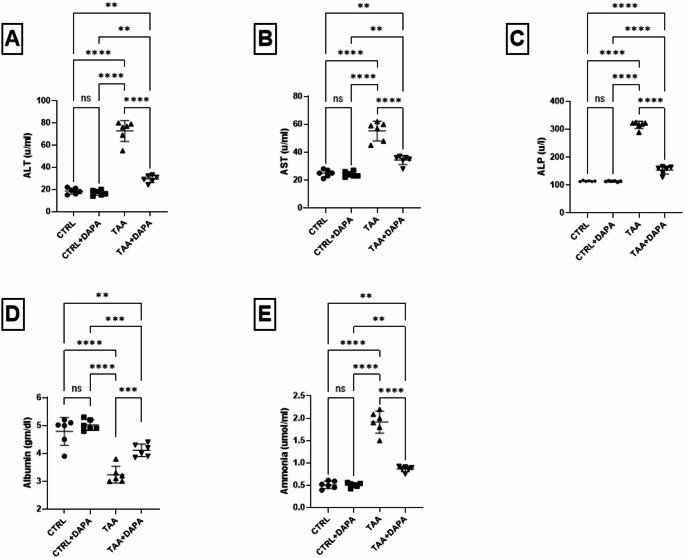
Effect of dapagliflozin (1 mg/kg, p.o.) on (**A**) ALT, (**B**) AST, (**C**) ALP, (**D**) albumin, and (**E**) Ammonia in TAA-induced (300 mg/kg, i. p.) hepatic encephalopathy in rats. Values are expressed as mean ± SD (*n* = 6). Statistical analysis was performed using one-way ANOVA followed by Tukey’s post-hoc test, with a significant value set at ns: non-significant, * *P* < 0.05, ** *P* < 0.01, ***P < 0.001, and **** *P* < 0.0001

Compared to the control group, TAA elevated ALT, AST, ALP and ammonia serum levels by about 3.92, 2.22, 2.76 and 3.76 folds, respectively. Furthermore, a notable reduction in albumin levels by 1.48 folds was observed Fig. 2**(A-E)**.

After DAPA dosing period, the serum levels of ALT, AST, ALP and ammonia were reduced by 59.40%, 37.77%, 51.33% and 54.92%, respectively compared to TAA group. Additionally, albumin levels were normalized by 26.98%. Figure 2(A-E).

### Histopathological findings of hepatocytes

**Fig. 3:**
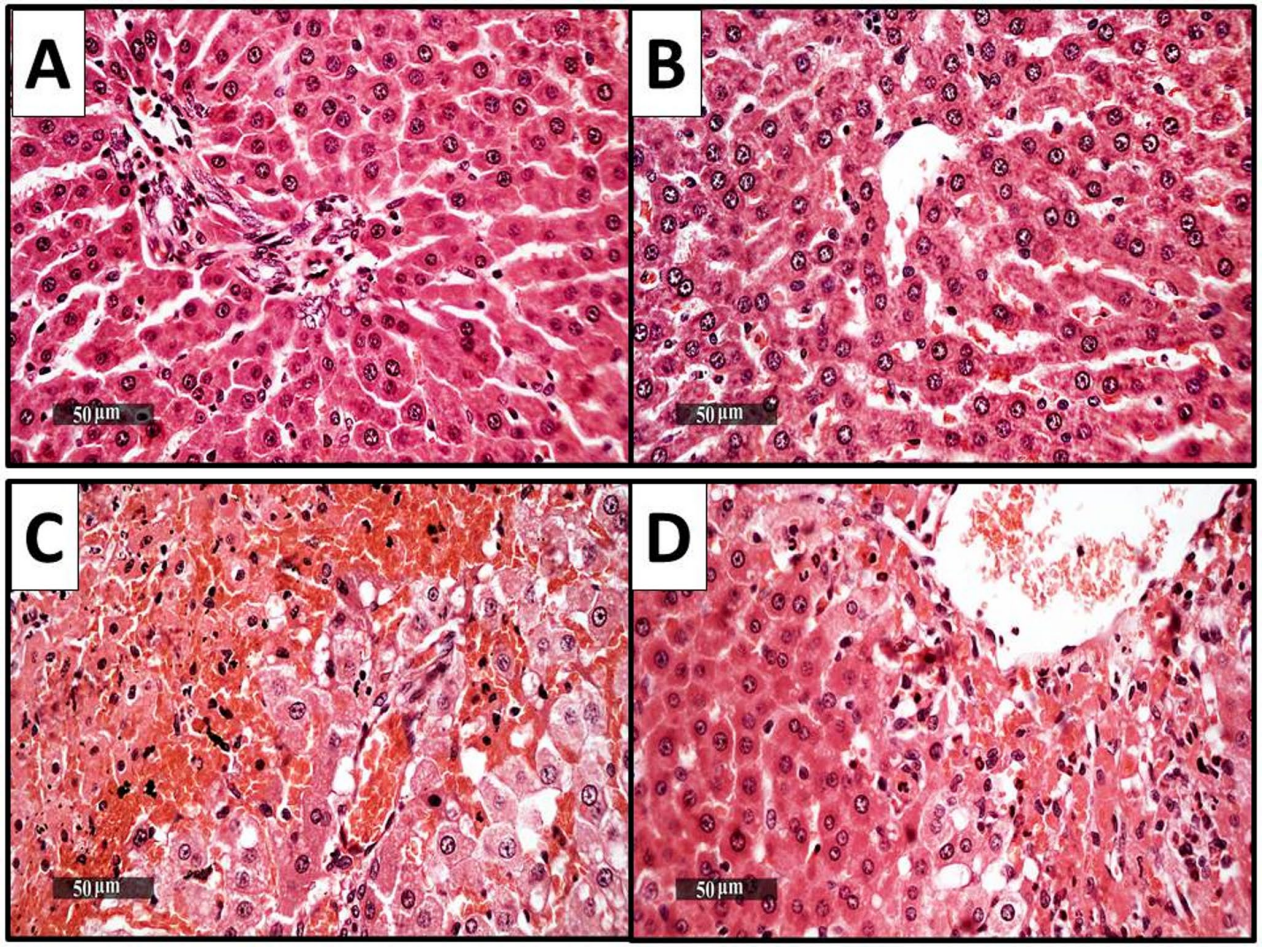
Histopathological finding of the liver in the different experimental groups. (**A**) Control group, (**B**) CTRL + DAPA group, (**C**) TAA group, (**D**) TAA + DAPA group (H&E, x40)

Figure 3 illustrates the outcomes of histopathological liver changes compared to the control group, which showed typical morphological characteristics of the rat liver parenchyma, including typical, well-organized hepatocytes with unchanged sub-cellular specifics. Hepatocyte degeneration, intact hepatic vessels, and hepatic sinusoids were rare sporadic occurrences, but no abnormal changes were discovered **(**Fig. 3A**)**. The samples from the CTRL + DAPA group revealed nearly intact hepatic parenchymal histology, with many records of hepatocytes that appeared to be in good condition and had intact subcellular details and only a few sporadic records of degenerated hepatocytes and healthy vasculature **(**Fig. 3B**)**. The model (TAA) samples revealed extensive pericentral-bridged hepatocellular necrosis mixed with extravasated blood. In addition to the hepatic vasculature and sinusoids being severely dilated, moderate to severe inflammatory cell infiltrates were also present **(**Fig. 3C**)**. Dapagliflozin’s moderate hepatoprotective efficacy was evident in model samples, alternating between clearly intact hepatocyte cords, milder inflammatory cell infiltrates, and minimal perivascular hemorrhagic patches. However, persistent moderate vascular congestion was also observed **(**Fig. 3D**)**.

### Evaluation of brain antioxidant capacity

**Fig. 4:**
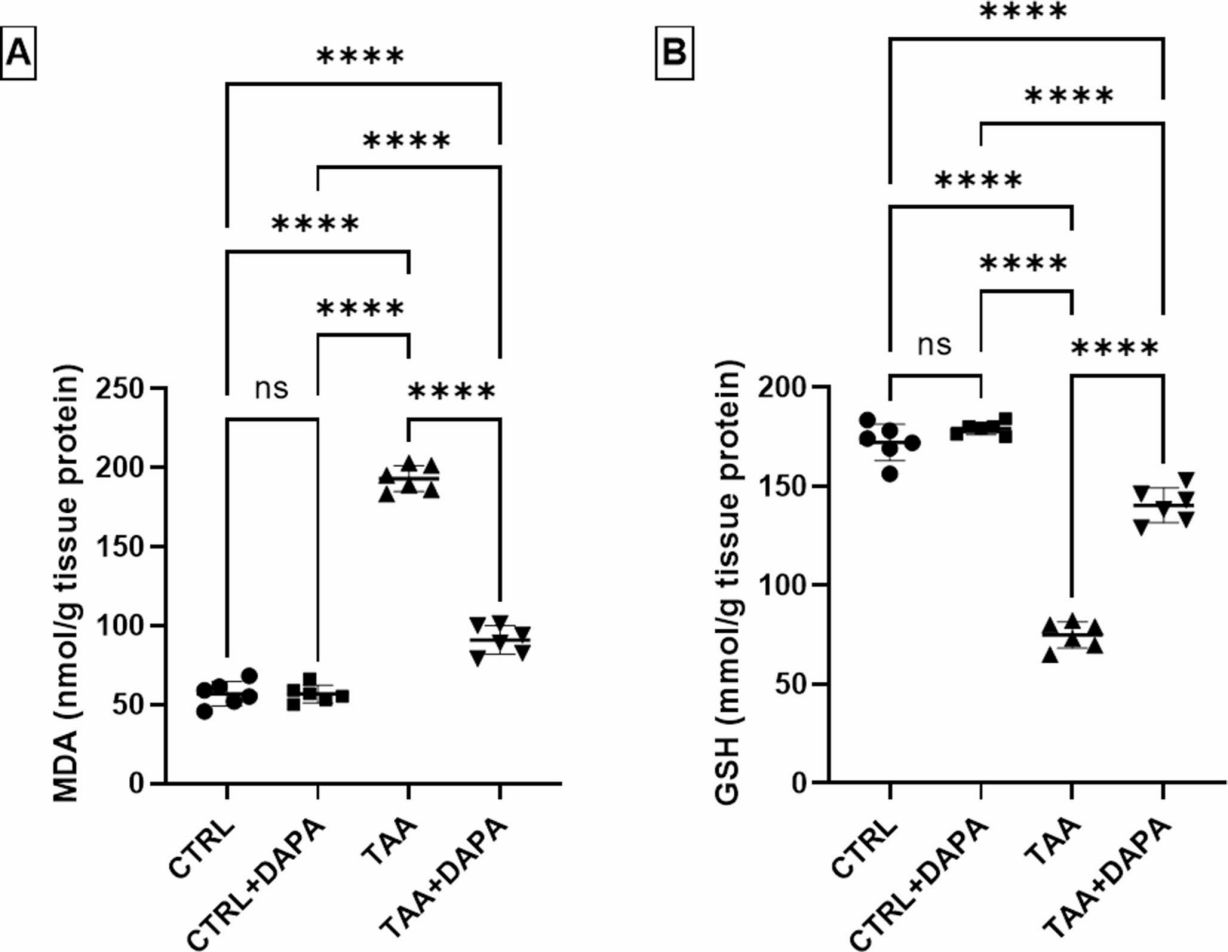
Effect of dapagliflozin (1 mg/kg, p.o.) on (**A**) MDA and (**B**) GSH in TAA-induced (300 mg/kg, i. p.) hepatic encephalopathy in rats. Values are expressed as mean ± SD (*n* = 6). Statistical analysis was performed using one-way ANOVA followed by Tukey’s post-hoc test, with a significant value set at ns: non-significant, * *P* < 0.05, ** *P* < 0.01, ***P < 0.001, and **** *P* < 0.0001

As depicted in Fig. 4 (A-B), initiation of HE *via* TAA resulting in depletion of brain antioxidant capacity proved by reduction of glutathione brain content by about 2.29 folds and significant increase in malondialdehyde brain content by 3.39 folds compared to control group. However, DAPA administration restored the brain antioxidant activity evidenced by elevation of glutathione brain content by 87.53% and reduction of malondialdehyde brain content by 52.86% compared with TAA group.

### Assessment of TLR4 and Notch1 gene expression

**Fig. 5:**
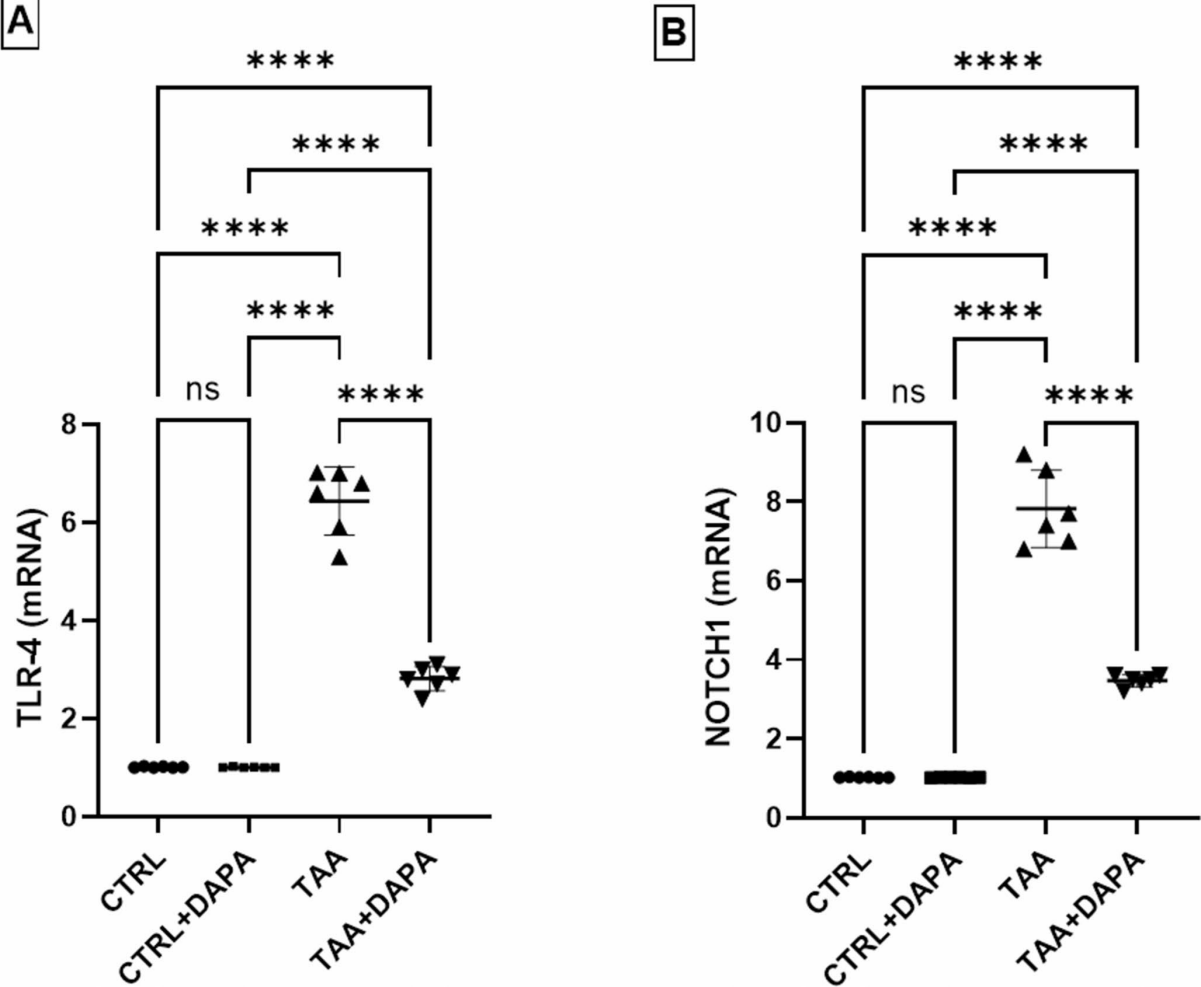
Effect of dapagliflozin (1 mg/kg, p.o.) on Toll receptor-4 (TLR-4) (**A**) and Notch1 receptor (**B**) gene expression in TAA-induced (300 mg/kg, I.P.) hepatic encephalopathy in rats. Values are expressed as mean ± SD (*n* = 6). Statistical analysis was performed using one-way ANOVA followed by Tukey’s post-hoc test, with a significant value set at ns: non-significant, * *P* < 0.05, ** *P* < 0.01, ***P < 0.001, and **** *P* < 0.0001

Figure 5A demonstrated that, rats with HE achieved by TAA showed a notable elevation in gene expression of Toll receptor-4 by about 6.37 folds compared to control group. DAPA significantly halved TLR4 gene expression.

Notch1 gene expression showed a significant elevation in the TAA group by 7.64 folds compared to control. However, DAPA showed a notable decrease in Notch1 mRNA levels by 55.65% compared to TAA group, indicating its potential to curb the Notch signaling pathway **(**Fig. 5B**)**.

### Evaluation of brain hippocampal tissues inflammatory cytokines

**Fig. 6:**
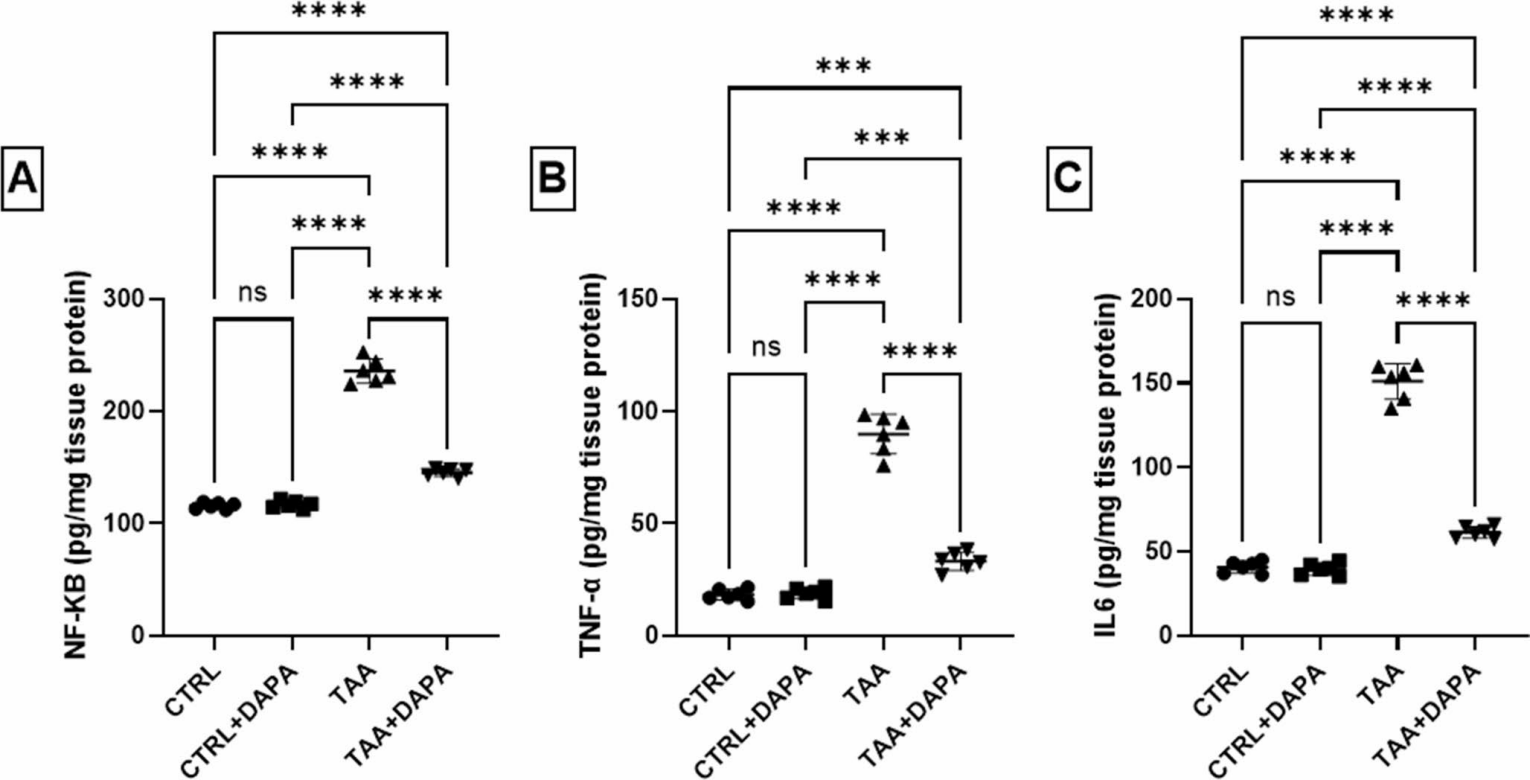
Effect of dapagliflozin (1 mg/kg, p.o.) on (**A**) NF-κB, (**B**) TNF-α, and (**C**) IL-6 in TAA-induced (300 mg/kg I.P.) hepatic encephalopathy in rats. Values are expressed as mean ± SD (*n* = 6). Statistical analysis was performed using one-way ANOVA followed by Tukey’s post-hoc test, with a significant value set at ns: non-significant, * *P* < 0.05, ** *P* < 0.01, ***P < 0.001, and **** *P* < 0.0001

The results presented in Fig. 6**(A-C)** revealed that, over expression of both TLR4 and Notch1 in TAA induced HE rats resulted in notable elevation in the brain content of NF-κB by 2.03 folds, TNF-α by 4.89 folds, and IL-6 by 3.70 folds compared to healthy rats. DAPA hindered the expression of both TLR4 and Notch1 leading to a marked reduction in NF-κB content by 38.24%, TNF-α content by 63.10%, and IL-6 content by 59.35% compared to TAA rats.

### Histopathological findings of hippocampus (CA3)

**Fig. 7:**
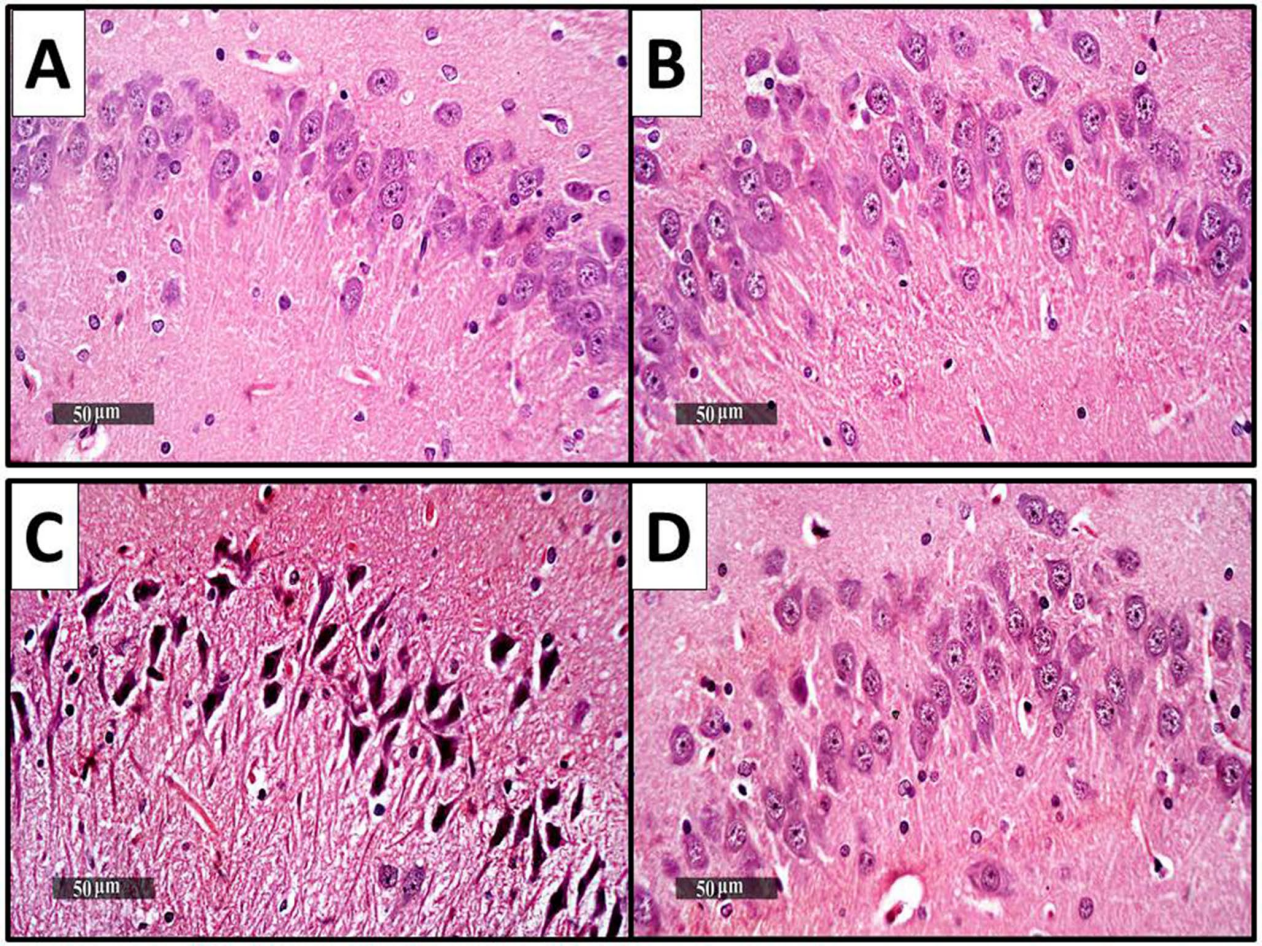
Histopathological finding of the brain in the different experimental groups. (**A**) Control group, (**B**) CTRL + DAPA group, (**C**) TAA group, (**D**) TAA + DAPA group (H&E, x40)

As shown in Fig. 7, the histopathological outcomes of the hippocampal tissues obtained from the control group revealed typically organized morphological characteristics of the hippocampal layers with abundant figures of apparent typical pyramidal neurons, demonstrating intact nuclear and cytoplasmic details. The intercellular brain matrix was intact without abnormal cellular infiltration **(**Fig. 7A**)**. The CTRL + DAPA group samples showed regularly organized morphological features of the hippocampal layers and cellular elements without abnormal recorded alterations **(**Fig. 7B**)**. TAA samples showed significant diffuse neuronal damage and loss, with many figures of shrunken, pyknotic, and angular neurons with indistinct subcellular details accompanied by moderate perineuronal edema, as well as significantly higher numbers of neuronophagia with reactive glial cell infiltrates **(**Fig. 7C**)**. DAPA-treated model samples demonstrated neuroprotective efficacy, with many apparent intact neurons. The minimal sporadic degeneration of neurons and milder persistent records of reactive glial cell infiltrates are shown **(**Fig. 7D**)**.

### Immunohistochemical assessment of cleaved Caspase-3 in the hippocampal tissues

**Fig. 8:**
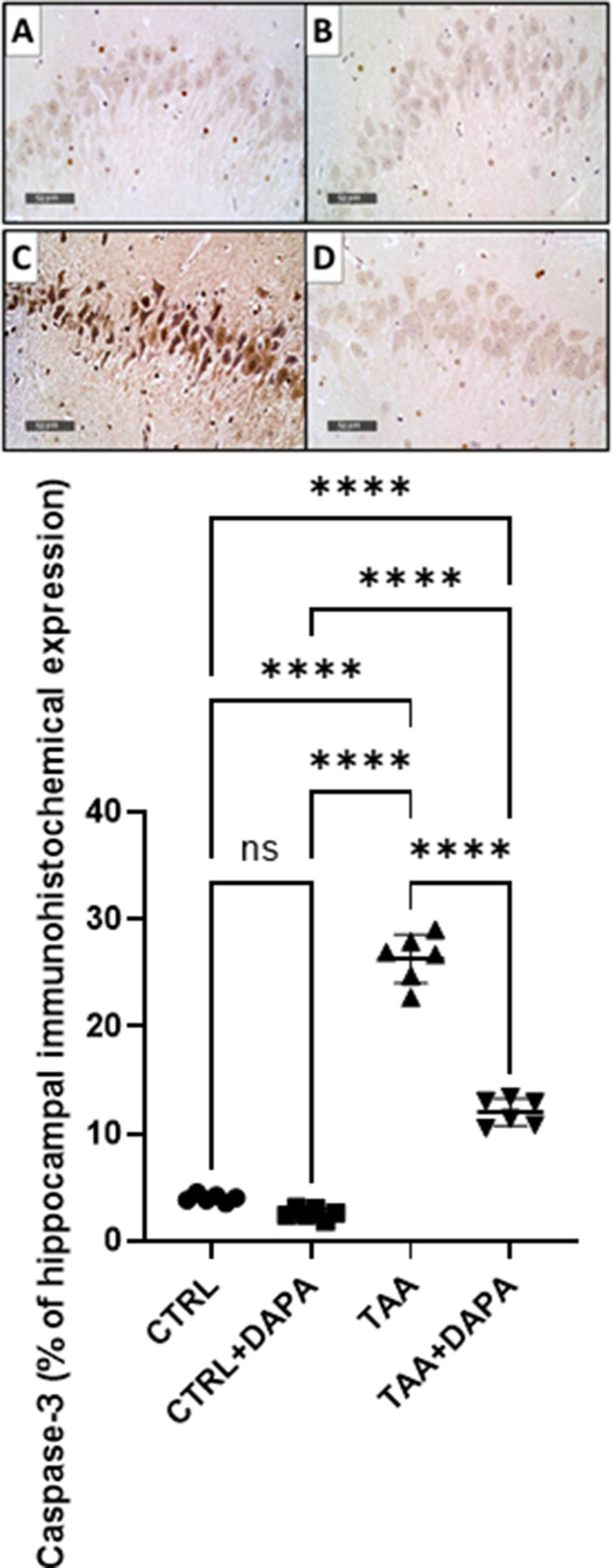
(A) Qualitative microscopic images of Caspase-3 immunostainnig in the brain tissues of different experimental groups. (**A**) Control group: showing weak immunoreactivity, (**B**) CTRL + DAPA group: showing weak immunoreactivity, (**C**) TAA group: showing intense immunopositive neurons, (**D**) TAA + DAPA group: showing marked reduction in the number of immunopositive neurons. (B) Quantitative analysis of the effect of dapagliflozin (1 mg/kg, p.o.) on Caspase- 3 protein expression in TAA-induced (300 mg/kg, I.P.) hepatic encephalopathy in rats. Values are expressed as the mean ± SD (*n* = 4). Statistical analysis was performed using one-way ANOVA followed by Tukey’s post-hoc test, with a significant value set at ns: non-significant, * *P* < 0.05, ** *P* < 0.01, ***P < 0.001, and **** *P* < 0.0001

The qualitative immunohistochemical assessment of Caspase-3 protein expression in the different experimental groups are summarized in Fig. 8A. Caspase 3 immunopositive glial cells and neurons were identified through its dark brown stain of both nucleus and cytoplasm. Minimal immunoreactivity for caspase-3 was observed in the brain tissue sections of control rats. Figure 8B demonstrated quantitatively that, induction of He by TAA resulted in significant increase in percentage of cells that immunopositive for Caspase 3 staining by 6.63 folds versus control rats. However, DAPA showed notable reduction in immunopositive cells percent for Caspase 3 staining by 54.41% against TAA group.

## Discussion

The current research, introduces a novel insight of dapagliflozin (DAPA) on cognitive and motor functions in rat model of hepatic encephalopathy (HE) induced by thioacetamide. It proved that DAPA improved learning and coordination in HE rats, as evidenced by reduction in escape latency of MWM test and elevated final falling-off time in Rotarod test. Moreover, DAPA ameliorated the hepatic and brain damage caused by TAA, as indicated by reduced serum levels of hepatic bio-markers, ammonia, and oxidative stress markers along with improved histopathological changes. Furthermore, DAPA’s possible mechanisms of action were investigated and suggested modulation of the TLR4/Notch1/NF-κB inflammatory pathway and reduction of apoptosis in the liver and brain tissues.

Thioacetamide induced HE was validated by impaired learning and coordination, as evidenced by increased escape latency in MWM test and reduced final falling-off time in Rotarod test. These findings support earlier research that reported similar behavioral deficits associated with HE induced by TAA in rats (Mustafa et al. 2013; El Khiat et al. 2022). This can be attributed to elevated ammonia levels in the brain, which activate astrocytes and microglia, trigger neuro-inflammation and increase gamma-aminobutyric acid (GABA) levels in the cerebellum (Hernandez-Rabaza et al. 2016). DAPA oral administration (1 mg/kg) to He rats for 28 consecutive improved cognitive and motor functions, as indicated by decrease in escape latency of MWM test and increase in final falling-off time of Rotarod test. These outcomes align with prior research that has displayed DAPA’s favorable effects on brain function in several models of neurological illnesses (Arab et al. 2021; El Khiat et al. 2022). Recently, DAPA neuroprotective effect has been investigated in numerous studies. Chen and colleagues elaborated that DAPA neuroprotective mechanism is multifactorial; and can be attributed to its antioxidant, anti-inflammatory, antiapoptotic, autophagic boosting and acetylcholinesterase suppressing effect; the latter effect improves cognitive function (Chen et al. 2024). DAPA’s anti-inflammatory effect was justified by the decrease in inflammatory mediators, M2 macrophage polarization, JAK2/STAT1 and NLRP3 inflammasome suppression. Moreover, DAPA advances endothelial performance, hinders remodeling and possesses a protective impact on the neurovascular unit, blood-brain barrier, pericytes, astrocytes, microglia, and oligodendrocytes. Furthermore, DAPA could repair the circadian rhythm of mTOR activation (Nguyen et al. 2020; Pawlos et al. 2021). Also, DAPA was proved to build-up in the seizing focus and stop seizures *via* regulation of neural electrophysiology (Erdogan et al. 2018; El-Safty et al. 2022; Donoiu et al. 2023).

The present research documented TAA hepatoxic effect by evaluating the serum levels of hepatic enzymes, which serve as a valuable indicator of liver cell damage and dysfunction (Afifi et al. 2018; Zheng et al. 2019). Both AST and ALT levels prominently increased upon treatment with TAA, which indicates severe hepatic injury (Mustafa et al. 2013). DAPA counteracted TAA induced rise of hepatic enzymes serum levels, reflecting its potential hepatoprotective effect (He et al. 2022).

To extend the current investigation, the effect of TAA and DAPA on ammonia metabolism was assessed. Ammonia is normally detoxified by the liver into urea and then excreted by the kidneys. However, in case of compromised liver, ammonia accumulates in the blood and reaches the brain, where it causes astrocyte swelling, cerebral edema, and neurological dysfunction; as a result glutamine osmotic effects (Norenberg 2003; Skowrońska and Albrecht 2013). Hyperammonemia neurotoxicity post hepatic affection can be attributed to the presence of mitochondrial ammonia (MA) delivered by glutamine released from glutamine synthetase (GS) ammonia clearing pathway. MA disturb the mitochondrial homeostasis; increase its oxidative stress level leading to astrocytes malfunction as well as inflammation and cerebral oedema due to the osmotic effects of glutamine (Albrecht and Norenberg 2006; Butterworth et al. 2009; Dabrowska et al. 2018). Furthermore, glutamine synthetase is very well associated to the metabolism glutamate and gamma aminobutyric acid (GABA), implicating a key role for this enzyme in neurotransmission (Zhou et al. 2020).

Herein, TAA administration significantly increased serum ammonia levels and decreased blood urea levels, indicating impaired ammonia detoxification and urea synthesis. Studies have shown that patients with HE tend to have systemic high ammonia levels (Heidari et al. 2016). In the current study, DAPA effectively counteracted the TAA induced hyperammonemia; suggesting that DAPA can help improve liver function and restore nitrogen metabolism balance.

It is well established that hyperammonemia results in immense release of reactive oxygen and nitrogen species (ROS and RNS), leading to elevated oxidative stress (Reddy et al. 2004). This stress damages neurons *via* lipid peroxidation, protein oxidation, and DNA damage. Additionally, it ruins the antioxidant defence capacity (Häussinger and Görg 2010; Rama Rao et al. 2014; Alomar and Al-attar 2019).

In harmony with previous studies, the current results demonstrated the impact of TAA and DAPA on brain oxidative stress and antioxidant defense; where TAA intoxication resulted in depletion of brain antioxidant capacity proved by reduction of glutathione brain content and significant increase in malondialdehyde brain content (Abdel Salam et al. 2010; Jain et al. 2011; Arauz et al. 2013; ElMahdy et al. 2020). DAPA was associated with the restoration of brain antioxidant activity evidenced by elevation of glutathione brain content and reduction of malondialdehyde brain content compared to those in TAA group. These outcomes adhere with the findings of Arab and colleagues (2021) that demonstrated the antioxidant features of DAPA (Arab et al. 2021).

To explore the mechanistic approach of DAPA neuroprotective impact, Toll receptor-4 (TLR4), which mediates macrophages activation and cytokines generation was assessed (Yuk and Jo 2011). Results revealed a marked up regulation of TLR4 expression in hippocampus tissue of TAA treated group, indicating the TLR4 involvement in the recognition and response to TAA-induced hepatic injury. This is consistent with previous reports that showed that hyperammonemia following hepatic insult, induces oxidative imbalance along with up-regulation of TLR4 expression. Thus, TLR4 acts as an antenna of inflammation and oxidative pressure caused by TAA and ammonia in brain tissue (Sinke et al. 2008; Lucas and Maes 2013; Jayakumar et al. 2015).^,^DAPA significantly attenuated the gene expression of TLR4 in hippocampus, suggesting that it reduced inflammation and oxidative stress caused by TAA and ammonia; aligned with the findings of Abdollahi and colleagues (Abdollahi et al. 2022).

Nuclear Factor Kappa B, is a master player in neuroinflammation (Pizzi et al. 2009; Harari and Liao 2010). TLR4 activation triggers NF-κB downstream signaling mastering both inflammation and immunity; resulting in astrocyte inflammation and cerebral edema. The produced cytokines also control the expression and activity of Nitric-oxide synthase, that produces the neurotoxic Nitric-oxide (Lu et al. 2020).

In harmony with recent studies, TAA administration significantly elevated serum levels of TNF-α, IL-6, IL-1β, and NO, revealing a systemic inflammatory reaction and nitrosative stress (Mohsen et al. 2021; Shaker et al. 2021). In contrast, DAPA demonstrated a marked reduction in the levels of these inflammatory mediators, indicating that it successfully inhibited the NF-κB pathway and its downstream effects (ElMahdy et al. 2020).

The Notch signaling is the second inflammatory controlling pathway explored in the current research (Chastagner et al. 2017; Steinbuck and Winandy 2018). It is essential in brain growth and function, particularly in the hippocampus. However, deviant activation of Notch pathway is proved as a contributor to neuroinflammation and neurodegeneration post brain insult (Grandbarbe et al. 2007; Albéri et al. 2010). In the present study TAA significantly elevated Notch1 receptor gene expression in the hippocampus of rats. Notch1 activation induced NF-Κb, initiating the gene expression of pro-inflammatory cytokines resulting in production and release of TNF-α, IL-1β, IL-6, and NO, leading to additional brain damage (Thomas 1992; Neumann et al. 2009; Sheng et al. 2018; Wu et al. 2018). DAPA significantly reduced the gene expression of Notch-1, NF-κB and TLR4 in the hippocampal tissue, implying its neuroprotective effects. Also, it reduced the release of downstream inflammatory cytokines as TNF-α and IL-6 (Faridvand et al. 2022; Li et al. 2022).

Apoptosis is another studied mechanism of neuronal destruction in HE, which is mediated by caspase-3 (Shabrang et al. 2017). TAA revealed a notable rise in Caspase3 protein expression in the brain, indicating an amplified level of neuronal apoptosis. This may be associated with the elevated oxidative stress and stimulation of TLR4 and Notch1 signaling pathways induced by TAA and ammonia. Recent studies have reported similar neuronal apoptosis findings in experimental HE models (Ferah et al. 2022; Okkay et al. 2022). DAPA markedly reduced the protein expression caspase-3 in the brain, suggesting a neuroprotective effect that may involve inhibiting ROS production and apoptotic pathways triggered by TLR-4 and Notch1 in HE rats. This is in line with preceding studies that demonstrated anti-apoptotic effects DAPA in diabetic rat models (El-Safty et al. 2022).

Throughout the current research, histopathological findings of liver and hippocampus tissue (CA3) from diseased rats revealed significant hepatocellular necrosis, astrocyte swelling, neuronal damage, and markers of HE, demonstrating morphological anomalies arising from hyperammonemia, depletion of antioxidant capacity, neuronal inflammation, and apoptosis. These outcomes are aligned with those of previously mentioned (Abdelaziz et al. 2015). DAPA treatment significantly ameliorated the morphological changes in both liver and hippocampus tissue, indicating its hepato-protective and neuro-protective effects in HE rat model initiated by TAA. These findings align with past research indicating that DAPA has favorable effects on liver and brain health in different experimental models (ElMahdy et al. 2020; Saleh et al. 2025).

Based on the observed results, DAPA is a promising pharmacological tool that can alter the prognosis of HE; this fact mandates further mechanistic studies in different neurological disorders to uncover its real potential. Undeniable limitations include missing experiments to study DAPA’s toxicity, kinetics and dynamics as well as other unassessed apoptotic indicators.

## Materials and methods

### Animals

The study utilized Wistar albino male rats with an average weight ranging from 180 to 220 g, which were procured from the Laboratory Animal House of the NRC in Cairo, Egypt. The four Rs principle was applied in the current study (Kiani et al. 2022). Rats were adapted for a week at the faculty of pharmacy, Cairo university animal house before starting the study. They were kept under controlled environmental conditions 12-hr light/12-hr dark cycle, room temperature, and a 60%−10% humidity level. They had unlimited access to standard rodent chow and purified water throughout the trial. The study protocol received approval from the faculty of pharmacy Ethical Committee for Animal Experimentation at Cairo University. These protocols adhered to the National Institutes of health of the United States regulations for care and use of lab. Animals (NIH publication # 85 − 23, revised 2011). All the necessary steps to lessen the animals’ pain and suffering were strictly followed.

### Drugs and chemicals

Dapagliflozin and Thioacetamide were acquired from sigma (Saint Louis, Missouri, USA). All chemicals utilized are of high analytical quality. Distilled water was used to dissolve both DAPA and TAA. DAPA was orally administered and TAA was intraperitoneally injected.

### Induction of hepatic encephalopathy

The Hepatic Encephalopathy rat model was achieved by a single intraperitoneal dosage of TAA (300 mg/kg) dissolved in sterile distilled water according to Mladenović and others (Mladenović et al. 2014).

### Experimental design

Forty rats were assigned into four equal groups, as illustrated in Fig. 1. The groups received the following treatments: Group I (CTRL): Intraperitoneal administration of distilled water for 29 days, serving as the normal control group; Group II (DAPA + CTRL): Oral administration of DAPA (1 mg/kg) (Millar et al. 2017) for 28 days, followed by an intraperitoneal injection of distilled water on day 29; Group III (TAA): Oral administration of distilled water for 28 days, followed by a single intraperitoneal injection of TAA (300 mg/kg) (Hernandez-Rabaza et al. 2016) on day 29 to induce experimental hepatic encephalopathy; Group IV (TAA + DAPA): Oral administration of DAPA (1 mg/kg) for 28 days (Hassan et al. 2025), followed by an intraperitoneal injection of TAA (300 mg/kg) on day 29.

After finishing the dosing phase, behavioral parameters were assessed. 24 h after behavioral parameters evaluation; blood specimens were drawn from the retro orbital venous plexus while anaesthetized using 30% diluted isoflurane *via* inhalation for induction and maintenance; used as 1 ml volume of mixture for every 200 ml volume of jar (Nagate et al. 2007). Subsequently, they were subjected to sacrifice by cervical-dislocation under phenobarbitone anesthesia (40 mg/kg I.P.) (Oh and Naver, 2024). Parts of the liver were collected from all groups, brains were carefully withdrawn, and hippocampal tissues were collected on ice cold plates. For further analysis, specimens were divided into multiple sections. Sacrificed rats were frozen until incineration. The investigators were blinded to the simple identity for all assessments, while an independent experimenter performed sample coding and decoding.

### Behavioral parameters

#### Morris water maze performance

To evaluate the spatial memory and learning, Morris water maze test was carried out as previously mentioned (Othman et al. 2022). Throughout the testing phase, a qualified individual revealed the delay to the initial hop on the concealed platform (escape latency in seconds). In every trial one rat was placed in the water, facing the pool’s edge, at one of four possible starts. Rat’s arrival and stay at the platform for 10 s, was considered as trial end and latency time was documented. Rats were allowed to dry for one minute before the following tials (Gamal et al. 2025).

#### Rotarod test

To assess the motor co-ordination rotarod test was applied through an accelerating rod (Model # 7750; Ugo Basile, Italy) following the protocol explained by (Vijitruth et al. 2006). The apparatus measured 120 cm in length and 3 cm in diameter, rotated at 25 rpm. The apparatus comprises five identical chambers raised 30 cm over a soft, padded cushion positioned underneath it to circumvent any harm from the fall. Every rat’s basal falling time was noted, with time Limit of 5 min, and maintained at the end of the session (48 h. following the final dose of Thioacetamide). Following the baseline measurement, each rat was positioned on the device for a 4-minute testing session, and final falling time was determined. Each animal underwent five separate training trials, each lasting 4 min. Then the probe test was conducted for a maximum duration of 4 min, and the time taken for each animal to fall was estimated **(**Saleh et al. 2025).

### Serum biochemical analysis

Serum samples were gathered by blood specimen centrifugation at 3000ˣg and 4 °C for 20 min after extraction from the retro-orbital venous plexus. Aspartate aminotransferase (AST; Cat.no # 260001) and Alanine aminotransferase (ALT; Cat.no # 264001) levels were determined calorimetrically utilising the technique described by Reitman & Frankel, (1957). Serum Albumin (ALB; Cat.no # 211001) levels were measured using bromocresol green following the technique described by Hinton et al. (1990). Serum ammonia (Cat.no # 220 001) was measured using the procedure previously explained by Konitzer and Voigt (1963). All parameters were measured utilising diagnostic kits available commercially from Spectrum Diagnostics (Obour City, Egypt).

### Histopathological examination of hepatocytes and hippocampus (CA3)

Liver and brain hippocampal tissues were fixed in 10% neutral-buffered formalin for 72 h in 10% neutral buffered formalin. Followed by treatment with ethyl alcohol, then cleared in xylene, and finally infiltrated and implanted into embedding media of paraplast tissue (Leica Biosystems). Rotary microtome was used to cut a 5 μm thick serial liver tissue sections from different samples and fixed on glass slide. Following staining with Hematoxylin and Eosin, an expert histologist blindly examined the sections (Culling 1985).

### Evaluation of brain hippocampal antioxidant capacity

Brain lipid peroxide levels were estimated using the technique described by Ruiz-Larrea and colleugues (1994). The glutathione (GSH) content was assessed utilizing the procedure described by Beutler and others (1963).

### Assessment of brain hippocampal TLR4 and Notch1 gene expression

Real time PCR, was conducted adhering to the previously described protocol (Liu et al. 2018). Quantitative RT-PCR was performed on RNA extracts from brain hippocampal tissues representing all the tested groups. RNA extracts from hippocampus tissues were obtained *via* TRIzol-reagent (Takara Bio Inc., USA). The extracted RNA was quantified spectrophotometrically *via* a NanoDrop^®^ (*Thermo Fisher Scientific*, Wilmington, DE, USA). RNA purity was evaluated using the 260/280 ratio, and a value of ~ 2 were regarded as satisfactory pure. The sample concentration was attuned to 30 ng/µL. The extracted RNA was reversely transcribed to its complementary DNA through HiScript-2 Single Step quantitative Real time Polymerase Chain Reaction SYBR-Green kit (Takara Bio Inc., USA) in accordance with the manufacturer’s guidelines. RT-PCR was determined in the existence of SYBR-Green-I fluorescent dye. Finally, it was normalized to GAPDH-ribosomal RNA in order to calculate mRNA-relative abundance. Primer sequences utilized are presented in Table 1.

**Table 1:** Table 1 Note: This data is mandatory, Please provide

TLR-4	TLR4-For	CCA GAG CCG TTG GTG TAT CT
	TLR4-Rev	TCA AGG CTT TTC CAT CCA AC
Notch1	Notch1-For	AGA GCT TTT CCT GTG TCT GTC C
	Notch1-Rev	CGG TAC AGT CAG GTG TGT TGT

### Evaluation of brain hippocampal inflammatory cytokines

The assessment of Nuclear Factor-Kappa B (NF-κB), Tumor Necrosis Factor-α (TNF-α), and Interleukin-6 (IL-6) in rats brain hippocampal was conducted utilizing Rat ELISA kits. The specific kits used were as follows: NF-κB (MYBIOSOURCE, San Diego, California, USA; Cat.# MBS015549), TNF-α (CUSABIO., USA; Cat.# CSB-E11987r), and IL-6 (R&D Systems inc., Minnesota, USA; Cat.# R6000B).

### Immunohistochemical assessment of cleaved Caspase-3 in the brain hippocampal tissues

Immunohistochemical assessment of cleaved Caspase-3 was applied on 5 μm-thick paraffin-embedded tissue sections of the hippocampal region. Brain hippocampal samples were treated with Caspase-3 polyclonal Antibody (active/cleaved) [(NB 100-56113 Cat.# IMG-5700), 1:1000 - Novus Biologicals, USA], and the resulting sections were stained with a secondary antibody (Cat.# HAF017 - Novus Biologicals, USA) and DAB. The sections were dried, counterstained with hematoxylin, and prepared for microscopic analysis. Leica’s histological analysis application module was used to undertake a histological analysis of six non-overlapping fields. The mean percentage area of cleaved Caspase 3 immunohistochemical expression in the hippocampal region was determined (Mohamed and Magdy, 2017).

### Statistical analysis

The data are presented as the mean ± SD of 8 rats per group regarding behavioral parameters and 6 rats per group for biochemical analysis. However, for histological analysis, there were 4 rats per group. Comparison between groups was done using One-way analysis of variance (ANOVA) followed by Tukey’s post hoc test. Statistical significance was expressed as non-significant (ns), significant at *P* < 0.05*, *P* < 0.01**, *P* < 0.001***, and *P* < 0.0001****. Statistical analysis and generation of figures were conducted using GraphPad Prism version 9.1 for Microsoft windows operating system (GraphPad Software, San Diego, California, USA).

## Conclusion

Concisely, Dapagliflozin exerted a neuro-protective effect in Hepatic Encephalopathy rat model induced by Thioacetamide.This was proved by enhanced cognitive, motor functions, and morphological changes. The Neuro-protective effect of Dapagliflozin was mediated through the reduction of hyperammonemia, the enhancement of hepatic functions, inactivation of TLR4/Notch1/NF-κB pathway, and apoptosis inhibition.

## Supplementary Information

Below is the link to the electronic supplementary material.

Supplementary file1 (JPG 187 KB)

## Data Availability

No datasets were generated or analysed during the current study.
