# Supplementary figures and images for: TLR-4/Notch1/NF-κB pathway modulation by dapagliflozin: a novel mechanism for neuroprotection in hepatic encephalopathy
Source: Metab Brain Dis. 2025 Sep 8;40(7):260. doi: 10.1007/s11011-025-01681-z (PMC12417255; doi:10.1007/s11011-025-01681-z)

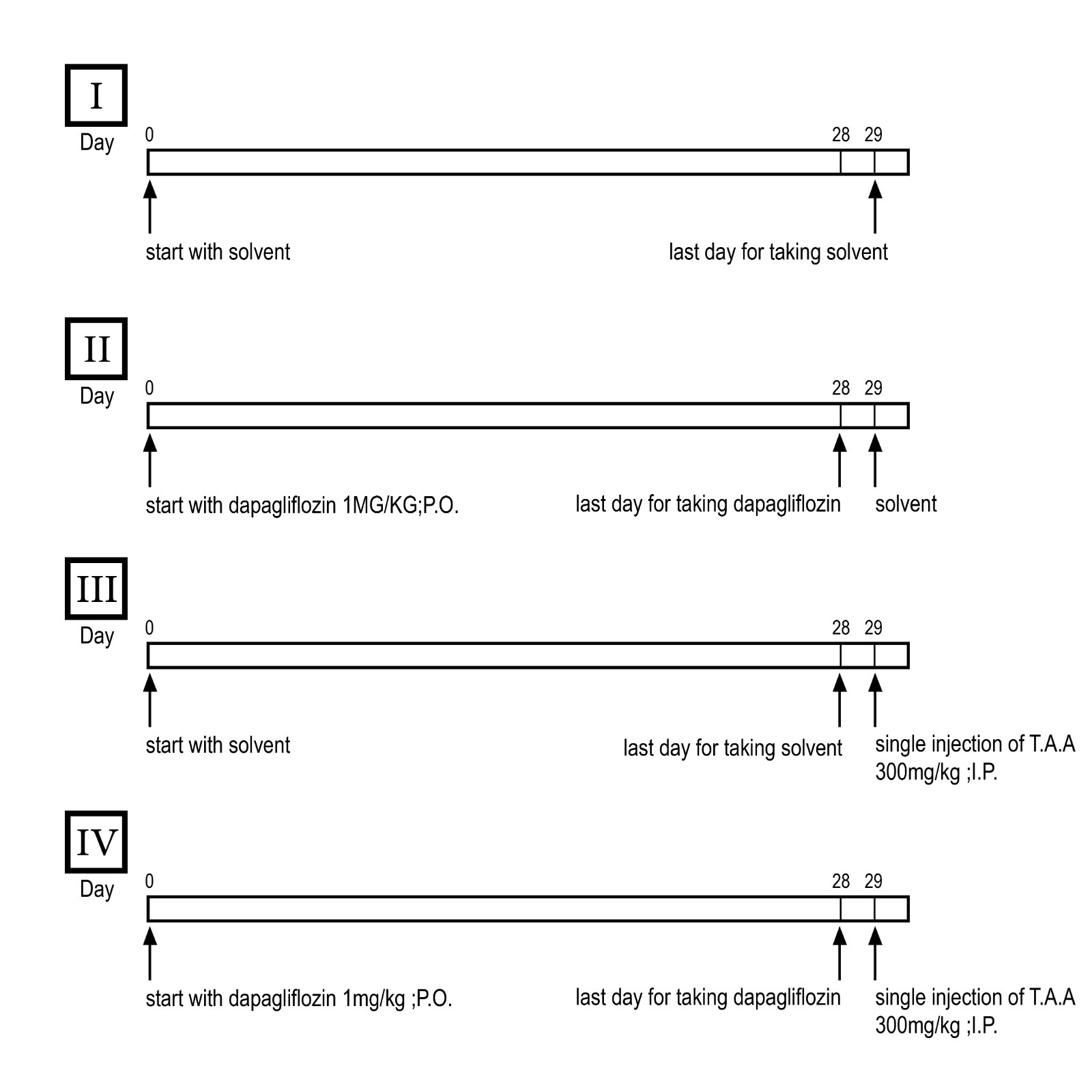

Supplement: Supplementary file 1 — Supplementary file1 (JPG 187 KB) [file 11011_2025_1681_MOESM1_ESM.jpg]
